# Regulatory mechanism and promising clinical application of exosomal circular RNA in gastric cancer

**DOI:** 10.3389/fonc.2023.1236679

**Published:** 2023-11-29

**Authors:** Ming Han, Mengyuan Zhang, Mei Qi, Yue Zhou, Fulong Li, Shengquan Fang

**Affiliations:** Department of Gastroenterology, Yueyang Hospital of Integrated Traditional Chinese and Western Medicine, Shanghai University of Traditional Chinese Medicine, Shanghai, China

**Keywords:** gastric cancer, exosomal circRNAs, epithelial-mesenchymal transition, chemotherapy resistance, biomarkers

## Abstract

Gastric cancer (GC) is one of the most common malignancies worldwide and the leading cause of cancer-related deaths. Exosomes are nanoscale extracellular vesicles secreted by a variety of cells and play an important role in cellular communication and epigenetics by transporting bioactive substances in the tumor microenvironment (TME). Circular RNA (circRNA) is a type of non-coding RNA (ncRNA) with a specific structure, which is widely enriched in exosomes and is involved in various pathophysiological processes mediated by exosomes. Exosomal circRNAs play a critical role in the development of GC by regulating epithelial-mesenchymal transition (EMT), angiogenesis, proliferation, invasion, migration, and metastasis of GC. Given the biological characteristics of exosomal circRNAs, they have more significant diagnostic sensitivity and specificity in the clinic and may become biomarkers for GC diagnosis and prognosis. In this review, we briefly describe the biogenesis of exosomes and circRNAs and their biological functions, comprehensively summarize the mechanisms of exosomal circRNAs in the development of GC and chemotherapy resistance, and finally, we discuss the potential clinical application value and challenges of exosomal circRNAs in GC.

## 1 Introduction

Gastric cancer (GC) is a common gastrointestinal malignancy with a high incidence and mortality rate. According to global cancer statistics from the International Agency for Research on Cancer (1), there were 1.09 million new cases of GC worldwide in 2020, making up 5.6% of all diagnosed cancer cases; of these, approximately 770,000 were related to GC deaths, accounting for 7.7% of all cancer-related deaths (2). GC is caused by a combination of factors, including pathogenic infections such as Helicobacter pylori and Epstein Barr virus, poor lifestyle habits such as unclean diet, smoking, high salt intake, consumption of large amounts of red meat and processed meat products, etc., which may increase the risk of GC to a certain extent (3). In addition, family inheritance and gene mutation are also significant factors leading to the development of gastric cancer (4). GC remains a significant problem with a heavy social and economic burden on human life and health (5), despite the current decline in GC incidence and mortality, as well as the further understanding and advances in the epidemiology and pathological mechanisms of GC (6–8). In the absence of sensitive and specific diagnostic markers as well as precise and effective therapeutic targets at an early stage, the early detection rate of GC and the effectiveness of treatment remain limited. Most of patients with gastric cancer are diagnosed at the advanced stage of the disease and are often accompanied by lymph node and peritoneal metastases (9). For these gastric cancer patients with metastases, the effect of surgical treatment is limited, chemotherapy is often resistant to drugs, and the effect of targeted and immune therapies is not satisfactory, resulting in a poor clinical prognosis (10). Furthermore, the heterogeneity of GC tumor tissue with genetic and epigenetic variations is a major source of complexity in GC treatment (11). Therefore, the search for highly sensitive and specific GC diagnostic markers and precise therapeutic targets is of great significance for the early diagnosis, treatment, and prognosis of GC. The tumor microenvironment (TME) is an essential site for tumor cell growth and development, and among the many components of the TME, exosomes are an indispensable part of it (12, 13).

Exosomes are extracellular vesicles between 30-150 nm in diameter with a phospholipid bilayer composed of various proteins, lipids and nucleic acids (14). The phospholipid bilayer protects protect the material within the exosome from removal or degradation. To maintain homeostasis and resist stress in the organism, exosomes carry out intercellular communication by delivering functional substances (15). Several recent studies have shown that exosomes are closely associated with human health and diseases, including cancer, neurological disorders, cardiovascular diseases, inflammatory diseases, and autoimmune diseases (16). Exosomes are involved in tumor development through multiple pathways including regulating tumor growth, invasion, migration, and angiogenesis (17). Exosomes and their contents have the potential to serve as diagnostic and prognostic biomarkers for cancer, as therapeutic targets for cancer, and as drug delivery vehicles for cancer therapeutics (18, 19).

Circular RNAs (circRNAs) are endogenous biomolecules in eukaryotic cells and are considered to be a member of the large family of non-coding RNAs. Unlike microRNAs (miRNAs) and linear RNAs, circRNAs have a unique ring-like covalently linked structure that makes them highly stable and more tolerant of degradation of their internal molecules by nucleic acid exonucleases (20). CircRNAs have multiple roles in tumor development, including regulation of tumor cell cycle and proliferation, regulation of cellular autophagy or apoptosis, angiogenesis, modulation of cellular energy metabolism, and evasion of tumor immune surveillance (21). An increasing number of studies have shown that circRNAs are widely present in exosomes and are involved in various pathophysiological processes. CircRNAs are delivered by exosomes to different receptor cells and contribute to tumorigenesis through local or distant regulation of interactions with receptor cells, as well as through regulation of epithelial-mesenchymal transition (EMT), invasion, metastasis, angiogenesis, and chemoresistance, and play a vital role in TME (13, 22, 23).

Exosomal circRNAs are currently very promising for research as diagnostic and prognostic biomarkers of GC. In this review, we briefly introduce the biogenesis and functional characteristics of exosomes and circRNAs, summarize the roles and mechanisms of exosomal circRNAs in GC progression, and explore their potential clinical applications in GC diagnosis and treatment.

## 2 Biogenesis and biological functions of exosomes and circRNAs

### 2.1 Biogenesis of exosomes

**Figure 1:**
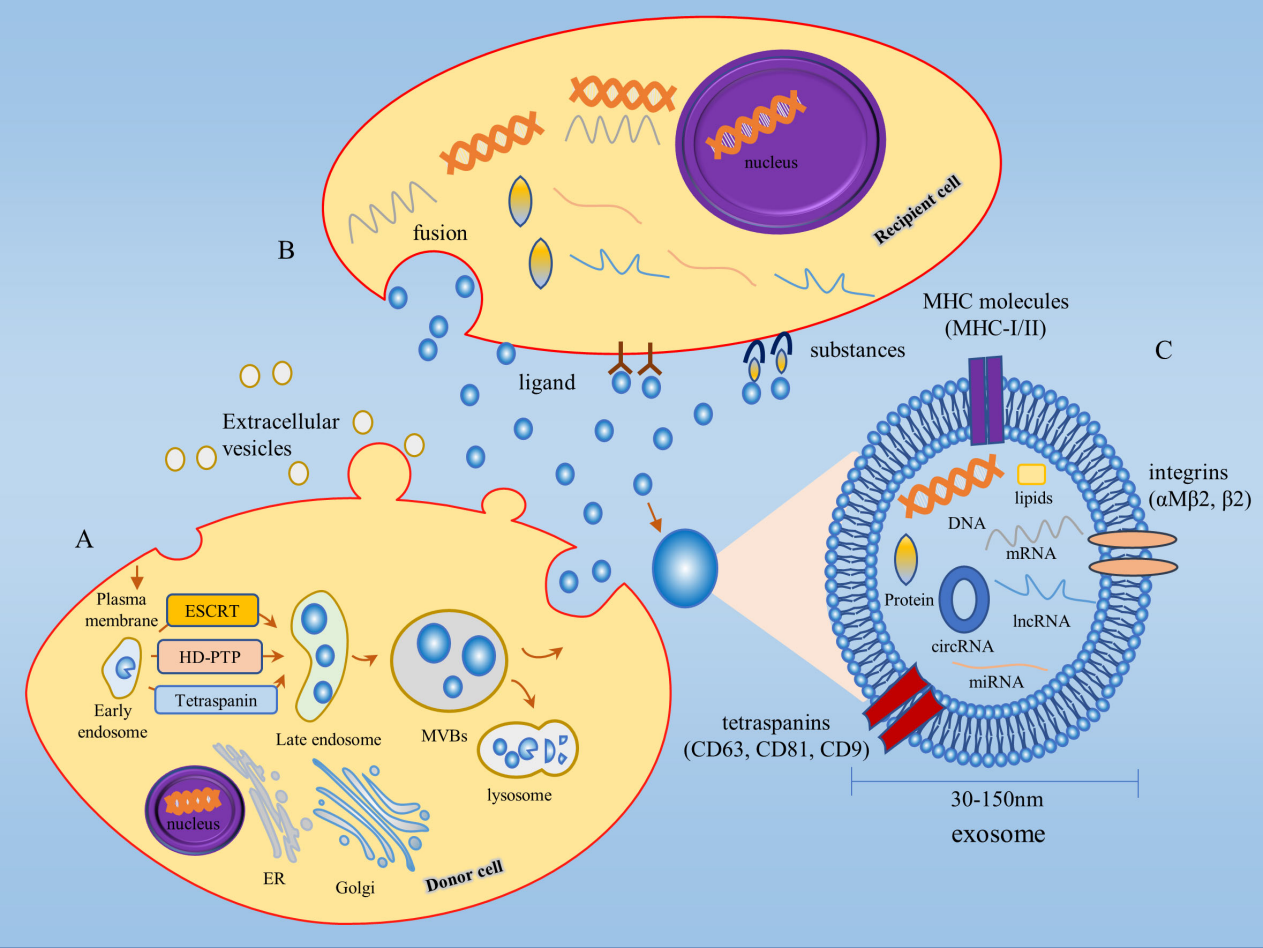
Biogenesis and biological function of exosomes. **(A)** Exosome biogenesis begins with endocytosis of the cell membrane, followed by the production of early endosomes, which mature to form MVBs. Some MVBs fuse with the PM and the ILVs are released outside the cell to form exosomes. ILVs production involves both ESCRT-dependent and ESCRT-independent pathways. **(B)** Exosomes exert their biological functions in three main ways: 1) ligand binding to receptors; 2) fusion; 3) substances of exosomes act on target cell surface receptors. **(C)** Exosomes are extracellular vesicles between 30-150 nm in diameter, consisting of proteins, lipids, DNA, mRNAs, miRNAs, lncRNAs, circRNAs, and other substances in a phospholipid bilayer structure. MHC molecules (MHC-I/II), tetraspanins (CD63, CD81, CD9), integrins (αMβ2, β2) and other protein molecules are distributed on the membrane of exosomes. MVBs, Multivesicular bodies; ILVs, Intraluminal vesicles; Golgi, Golgi apparatus; ER, Endoplasmic reticulum.

The biogenesis of exosomes begins with endocytosis of the cell membrane, where the plasma membrane (PM) budded into the cell to produce initial vesicles, or early endosomes, whose role is to sort the molecular material being endocytosed (24). The early endosomes mature into late endosomes, which encapsulate specific proteins, lipids, nucleic acids, and other materials to form multiple intraluminal vesicles (ILVs), the precursors of exosomes. Late endosomes continue to develop and mature to form multivesicular vesicles (MVBs), which are essential for exosome biogenesis and MVBs can dynamically communicate with other organelles such as the Golgi apparatus (Golgi), endoplasmic reticulum (ER), and autophagosomes through multiple pathways (25–27). Most MVBs fuse with lysosomes, resulting in the degradation of both MVBs and their contents, while a small proportion of MVBs fuse with the PM, resulting in the release of ILVs to form extracellular vesicles, also known as exosomes (28) (**Figure 1**).

Exosomes are highly heterogeneous since each stage of exosome biogenesis is mediated by many processes that depend on various molecular substances, cell types, and cellular microenvironments. The production of early endosomes and ILVs is regulated by a variety of mechanisms, the most studied being the endosomal sorting complex required for transport (ESCRT) dependent pathway and the ESCRT-independent pathway (15, 29). For the classical ESCRT-dependent pathway, ESCRT-0, -I, -II, -III subcomplexes and the ATPase VPS4 synergistically mediate ILV formation, whereas non-classical ESCRT-dependent pathways, such as the HD-PTP-dependent pathway and the Alix-dependent pathway, can recruit ESCRT-III and VPS4 to the MVB by recognizing specific membrane-bound molecular substances to promote ILV formation (30, 31). Among the ESCRT-independent pathways, the nSMase2-ceramide-dependent pathway has been well studied, in addition to lipid components such as the membrane protein caveolin 1, the membrane backbone protein flotillins, cholesterol, and tetraspanins also play a critical role in the formation of ESCRT-independent ILV (15, 32).

### 2.2 Biological functions of exosomes

In the 1980s, exosomes, extracellular vesicles isolated from sheep reticulocytes cultured *in vitro*, were thought to be a dumping ground for cellular metabolites and did not receive sufficient attention (33). The exosomes secreted by EBV-infected B lymphocytes were later found to induce T-cell responses by antigen presentation (34). Subsequent studies further revealed that RNA within exosomes could exchange genetic material between cells (35). Today an increasing number of studies have focused on the intracellular biological processes of exosomes, finding that they play a significant role in intercellular communication and are involved in cell differentiation, tumor immune response, tumor cell proliferation, migration, and invasion (36).

Exosomes contain proteins, lipids, sugar structures, metabolites, DNA, messenger RNAs (mRNAs), miRNAs, long non-coding RNAs (lncRNAs), circRNAs, and other substances (37). Several protein molecules are distributed on the membrane of exosomes, including MHC molecules (MHC-I/II), tetraspanins (CD63, CD81, CD9), and integrins (αMβ2, β2), of which tetraspanins can be used as specific markers for the isolation of exosomes (36) (**Figure 1**). The cellular state from which exosomes originate drives the contents of exosomes in a dynamic state of flux and consequently determines the function of exosomes (38). Exosomes exert their biological functions in three main ways firstly, ligands on the exosome membrane bind to receptors to transmit intercellular information; secondly, exosomes are extensively involved in material transport by fusing with target cells and releasing the specific components they carry into the recipient cells; finally, exosomes release substances from the body and act on the receptors on the surface of the target cells to complete the information transfer and thus produce biological effects (39, 40). Exosomes are widely distributed throughout the body and can be found in a variety of bodily fluids, such as blood, saliva, urine, milk, cerebrospinal fluid, malignant exudates (peritoneal fluid), and cell culture media. They transmit molecular signals through autocrine, paracrine, and endocrine mechanisms (29). Tumor cell-derived exosomes, known as texosome (TEX), are an essential component of the TME, and the number of TEX is much higher than that secreted by normal tissues. TEX regulates the tumor microenvironment and promotes tumor cell proliferation and migration, and tumor cells in turn contribute to tumor progression by regulating the biogenesis, composition, and function of exosomes (15). In the early stages of the disease, the isolation and purification of exosomes can be used for clinical assessment (41). As research on exosomes progresses, several databases on exosomes have been established, including the ExoCarta database (http://www.exocarta.org/), the Fudan University exosome database (http://www.exoRBase.org), and the Vesiclepedia database (http://microvesicles.org/index.html).

### 2.3 CircRNAs biogenesis

**Figure 2:**
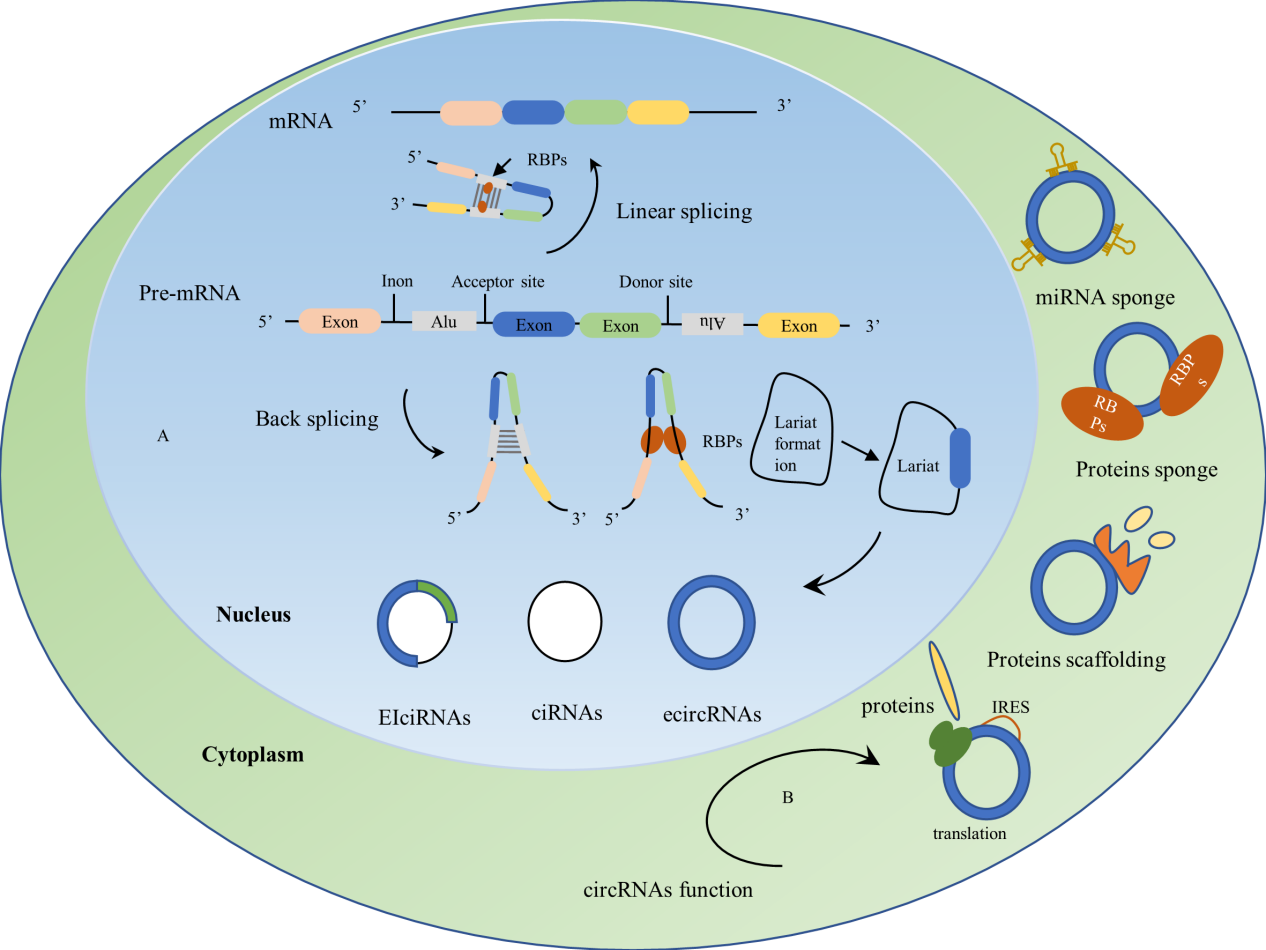
Biogenesis and biological functions of circRNAs. **(A)** Biogenesis of circRNAs: CircRNAs are derived from precursor mRNAs(pre-mRNAs), which form mRNAs by linear splicing and circRNAs by back-splicing. Mechanisms of circRNAs formation: RNA-binding proteins (RBPs) dimerize upon binding to flanking introns, upstream and downstream introns promote the formation of loop structures by the base complementary pairing of reverse repeat Alu elements, lariat formation during exon skipping and internal splicing of the lariat where the intron sequence is removed drives the formation of circRNAs. CircRNAs usually exist in three forms: exon circRNAs (ecircRNAs), intron circRNAs (ciRNAs), and exon-intron circRNAs (EIciRNAs). **(B)** Biological functions of circRNAs: 1) act as miRNA sponges; 2) act as protein sponges; 3) act as protein scaffolds by providing binding sites for protein assembly; 4) CircRNAs containing internal ribosome entry site (IRES) elements can be translated into proteins or peptides.

CircRNAs are a covalently closed-loop single RNA structure consisting of single or multiple exons, mostly derived from precursor mRNAs and expressed by known protein-coding genes (42, 43). Unlike the 3’ and 5’ ends of lncRNA structures, which are covalently linked, they are closed-loop structures with no tails at the 3’ and 5’ ends. CircRNAs are covalently linked at the flanking sites of the splice indicator to form a circRNA, the downstream splice donor site binds covalently to the upstream splice acceptor site, a phenomenon or process known as back-splicing. Unlike the normal form of splicing, this is a unique biological process for circRNAs (44). Despite the lower efficiency of back-splicing compared to linear splicing, circRNAs can accumulate in a time-regulated manner in a given cell while retaining their function for long periods without inactivation due to their high stability (45). CircRNAs have a longer half-life compared to linear RNAs and in some cases are 10 times more abundant than related linear RNAs (46). Besides, circRNAs contain all the products of selectively spliced linear RNAs, yet linear transcription does not contain some of the exons of circRNAs (47). The biogenesis of numerous circRNAs is influenced by combinations of cis-acting elements and trans-acting splicing factors, including heterogeneous nuclear ribonucleoproteins (hnRNPs) and long-repeat SR proteins containing serine and arginine amino acid residues (43). Mechanisms of circRNAs formation typically include RNA-binding proteins (RBPs) such as HQK and FUS binding to flanking introns after dimerization, which promotes circular splicing of circRNAs (48, 49); introns located upstream and downstream, which promote the formation of circular structures through the base complementary pairing of inverted repetitive Alu elements; lariat formation during exon skipping, which promotes the formation of circRNAs by internal splicing where intron sequences have been removed; moreover, intron escape debranching also contributes to the formation of circRNAs (22, 49). CircRNAs exist in three main forms (**Figure 2**), including exon circRNAs (ecircRNAs), intron circRNAs (ciRNAs), and exon-intron circRNAs (EIciRNAs), where ecircRNAs are mainly located in the cytoplasm, and ciRNAs and EIciRNAs are mainly located in the nucleus (40, 50) (**Figure 2**).

### 2.4 Biological functions of circRNAs

CircRNAs were first discovered by electron microscopy in the cytoplasmic fraction of eukaryotic cell lines (51) and were thought to be cellular ‘waste’ from abnormal splicing or by-products of abnormal RNA splicing (52, 53). The function of circRNAs was revisited when B Capel et al. discovered that Sry genes from the sex-determining region of the Y chromosome existed as circRNAs and played a significant role in the testis (54). As research progressed, circRNAs were found to act as a competitive endogenous RNA (ceRNA) or as a miRNA sponge (**Figure 2**), regulating gene expression by binding to miRNA, inhibiting miRNA binding to the 3’UTR of specific genes, and ultimately by triggering mRNA cleavage or mRNA translation inhibition (55). CircRNAs have a large number of binding sites for RBPs and can act as protein sponges while blocking the activity of RBPs (53). CircRNAs provide binding sites for protein assembly and act as protein scaffolds to form protein complexes (56). In addition, circRNAs can regulate the transcription and post-translation of parental genes to perform their biological functions (57, 58) (**Figure 2**). CircRNAs have been widely used in a variety of diseases, such as cardiovascular diseases (59), diabetes (60), neurological diseases (61), and cancer (21), with the application of circRNAs in cancer currently being a hot topic in oncology research. CircRNAs are abundantly expressed, highly stable, with tissue-restricted and cancer-specific expression patterns, and can be detected in liquid biopsies such as plasma, urine, and saliva. Dysregulated expression of circRNAs is involved in cancer development and plays a vital role in tumor cell proliferation, metastasis, and drug resistance (21).

## 3 Overview of exosomal circRNAs

It was found that circRNAs were highly enriched in exosomes and could be stably present in exosomes (62, 63), especially from TEX compared to exosomes secreted by normal cells, where the abundance of circRNAs was significantly increased. Dou et al. found that the number of circRNAs in exosomes secreted by colon cancer cell lines was greater than that of free circRNAs in colon cancer cells, but the relationship between circRNAs in colon cancer cells and their secreted exosomal circRNAs and their regulatory mechanisms were not clear (64). It has also been reported that circRNAs in exosomes are two times more abundant than circRNAs in parental cells and six times more abundant than linear RNAs compared to parental cells (63). The sorting of circRNAs into exosomes is regulated by changes in the levels of parental cell-associated miRNAs and transmits biological activity to recipient cells, thereby participating in intercellular communication (63). The nanosize and lipid bilayer structure of exosomes can prolong the circulation time of circRNAs and enhance their biological activity, thus exosomal circRNAs have both exosome-like transfer targeting characteristics and the original biological functions of circRNAs, resulting in more significant regulatory advantages (65). Several studies have discovered that tumor-specific circRNAs can be selectively packaged, secreted, and transported via TEX to participate in the regulation of TME, promoting or inhibiting tumor cell growth and metastasis, while exosomal circRNAs have more significant diagnostic sensitivity and specificity than free circRNAs in body fluids, and may become biomarkers for clinical diagnosis and prognosis (66).

## 4 Role and mechanism of exosomal circRNAs in GC

Exosomal circRNAs play multiple roles in GC, including promotion of GC proliferation, induction of EMT as well as invasion and migration of GC cells, mediation of GC angiogenesis, regulation of GC metastasis, modulation of chemoresistance, and radiosensitivity in GC **Table 1**.

**Table 1:** Role of exosomal circRNAs in GC.

CircRNAs	Mechanism		Role		Expression		Source		Characters	Ref
circ_0088300	KHDRBS3/circ_0088300/miR-1305/JAK/STAT		Enhanced proliferation, migration, and invasion		Up		CAF/Plasma		Carcinogenic	(67)
circNEK9	circNEK9/miR−409−3p/MAP7		Enhanced proliferation, migration, and metastasis		Up		Plasma		Carcinogenic	(68)
circFCHO2	circFCHO2/miR-194-5p/JAK1/STAT3		Enhanced Angiogenesis, proliferation, invasion, and stem cell properties		Up		Serum		Carcinogenic	(69)
circ-ITCH	circ-ITCH/miR-199a-5p/Klotho		Suppressed EMT, proliferation, migration, and invasion/synergistic effect in combination with cisplatin		Down		Serum		Anti-cancer	(70)
circNRIP1	QKI/circNRIP1/miRNA149-5p/AKT1/mTOR		Enhanced EMT, pulmonary and peritoneal metastasis/Regulates metabolism and autophagy		Up		Plasma		Carcinogenic	(71)
circUBE2Q2	circUBE2Q2/miR-370-3p/STAT3		Enhanced EMT, migration invasion, glycolysis		Up		Plasma		Carcinogenic	(72)
hsa_circ_0017252	As a miR-17-5p sponge		Reduced EMT/Suppressed macrophage M2-like polarization, EMT		Down		GC cell		Anti-cancer	(73)
circDIDO1	Suppressed PARP1 expression Enhanced RBX1-mediated ubiquitination and PRDX2 degradation; circDIDO1/miR1307-3p/SOSC2		Suppressed EMT, proliferation, migration, and invasion,		Down		RGD-modified		Anti-cancer	(74, 75)
circ670	–		Enhanced EMT, dryness of GCSC		Up		GCSC		Carcinogenic	(76)
circSHKBP1	circSHKBP1/miR-582-3p/HUR/VEGF Inhibition of ubiquitination of HSP90		Enhanced angiogenesis, proliferation, migration, invasion		Up		Serum		Carcinogenic	(77)
circ29/circ_0044366	As a miR-29a sponge		Enhanced proliferation, invasion, and malignant phenotype of HUVEC		Up		Plasma		Carcinogenic	(78)
circ_0001789	circ_0001789/miR-140-3p/PAK2		Enhanced EMT, angiogenesis		Up		GC cell		Carcinogenic	(79)
hsa_circ_0000437	hsa_circ_0000437/SRSF3/PDCD4; VEGF-C non-dependent HSPA2-ERK signaling pathway		Enhanced invasion, migration of HLECs/Enhanced lymphangiogenesis and LNM		Up		GC cell/Serum		Carcinogenic	(80)
circSTAU2	MBNL1/circSTAU2/miR-589/CAPZA1		Suppressed proliferation, migration, and invasion		Down		GC cell		Anti-cancer	(81, 82)
circ-RanGAP1	circ-RanGAP1/miR-877-3p/VEGFA		Enhanced proliferation, migration, and invasion		Up		Plasma		Carcinogenic	(83)
circRELL1	circRELL1/miR-637/EPHB3		Suppressed proliferation, migration, invasion, and anti-apoptotic capacity; Regulation of autophagy		Down		Plasma		Anti-cancer	(84)
circ_0063526	circ_0063526/miR-449a/SHMT2		Enhanced cisplatin resistance		Up		Serum		–	(85)
circ-PVT1	circ-PVT1/miR-30a-5p/YAP1		Enhanced cisplatin resistance Regulation apoptosis, invasion, and autophagy		Up		GC cell/Serum		–	(86)
circ-LDLRAD3	circ-LDLRAD3/miR-588/SOX5		Enhanced cisplatin resistance		Up		GC cell		–	(87)
circ_0000260	circ_0000260/mir-129-5p/MMP11		Enhanced cisplatin resistance		Up		GC cell/Serum		–	(88)
circ_0091741	circ_0091741/miR-330-3p/TRIM14/Dvl2/Wnt/β-catenin		Enhanced autophagy and oxaliplatin chemotherapy resistance		Up		GC cell		–	(89)
circPRRX1	circPRRX1/miR-596/NKAP		Interfere proliferation, migration, invasion, and radiosensitivity		Up		GC cell		Anti-cancer	(90)

### 4.1 Exosomal circRNAs regulate GC proliferation

Several studies have shown that exosomal circRNAs are involved in the regulation of GC proliferation. For example, Shi et al. revealed that cancer-associated fibroblasts (CAF) deliver circ_0088300 to GC cells via exosomes, and that overexpression of exosomal circ_0088300 promotes upregulation of anti-apoptotic protein Bcl-2 expression and downregulation of apoptotic proteins Bax, caspase 3 and caspase 9 expression, thereby enhancing malignant cell transformation *in vitro* and promoting GC cell proliferation. Mechanistically, the CAF-derived exosomal circ_0088300 regulates the JAK/STAT signaling pathway by sponging miR-1305 to promote the proliferation, migration, and invasion of GC cells. Moreover, RBP KHDRBS3 drives circ_0088300 encapsulation into exosomes and regulates circ_0088300 levels in exosomes to promote GC development (67). Similarly, another study demonstrated that plasma exosomal circNEK9 acts as a miR-409-3p sponge to upregulate MAP7 protein expression and promote GC cell proliferation, invasion, and metastasis (68). Zhang et al. found that circFCHO2 was upregulated in serum exosomes of GC patients. Functional assays suggested that circFCHO2 increased the proliferation, invasion, angiogenesis, and stem cell properties of GC cells. In a nude mouse xenograft tumor model, silencing of circFCHO2 attenuated GC cell growth and lung metastasis. Mechanistically, circFCHO2 acts as an oncogenic factor to promote GC progression by activating the JAK1/STAT3 signaling pathway by sponging miR-194-5p (69). Wang et al. reported that serum exosomal circ-ITCH expression was downregulated in GC patients and its expression level correlated with the depth of GC infiltration. Functional assays showed that overexpression of circ-ITCH inhibited EMT as well as proliferation, migration, and invasion of GC cells. Mechanistically, circ-ITCH acted as a sponge for miR-199a-5p and increased Klotho expression, thereby inhibiting GC invasion and migration. Interestingly, circ-ITCH was not detected in serum but could be detected in serum exosomes, suggesting that circ-ITCH could be enriched in exosomes. In addition, the combination of overexpression of circ-ITCH and cisplatin (CDDP) had a synergistic effect on GC cells (70).Thus, exosomal circRNA promotes or inhibits the malignant progression of GC through multiple mechanisms.

### 4.2 Exosomal circRNAs induce EMT, invasion, and migration of GC cells

EMT is the biological process by which epithelial cells are transformed into mesenchymal-like cells with stem cell-like characteristics, motility, and greater invasive capacity, and is an important procedure in the malignant progression of cancer. In the early stages of cancer, tumor cells have epithelial-like characteristics and acquire more mesenchymal properties as the tumor progresses, thus giving them the ability to invade and migrate (91). In the TME, paracrine types of cells can almost always release exosomes. Exosomes carry a variety of bioactive molecules, including circRNAs (92), which promote tumor EMT by activating the expression of EMT-inducible transcription factors (EMT-TF) or effector molecules, where EMT-TF can repress the expression of genes that maintain the epithelial state and promote the expression of genes in the mesenchymal cell state (93). During EMT, the expression of epithelial markers such as E-cadherin and ZO-1 is downregulated, and the expression of mesenchymal markers such as N-cadherin and vimentin is upregulated (94).

A large body of literature reports that exosomal circRNAs regulate EMT in GC cells and promote their invasion and migration. For instance, Zhang et al. found that circNRIP1 was highly expressed in GC tissues and cells, could be transported via exosomes secreted by GC cells, and promoted lung and peritoneal metastasis of GC via EMT. In addition, circNRIP1 knockdown inhibited GC cell proliferation, migration, invasion, and AKT1 expression levels, and mechanistically, circNRIP1 acted as a miR-149-5p sponge to induce GC cell metabolism and autophagy through regulation of the AKT1/mTOR pathway and promote GC progression via exosomal transport. RBP QKI was also found to promote the up-regulation of circNRIP1 in GC tissues. QKI may be a major regulator of circRNAs biosynthesis in EMT and may regulate the formation of circNRIP1 via a post-transcriptional pathway during GC development (71). Similarly, another study revealed that circUBE2Q2 is present in exosomes released from GC cells and is highly expressed in plasma exosomes. Exosomal circUBE2Q2 activates the STAT3 signaling pathway in an autocrine or paracrine manner and regulates the GC EMT process. CircUBE2Q2 promotes peritoneal metastasis as well as liver lymph node metastasis (LNM) in GC mice *in vivo*. Additionally, circUBE2Q2 inhibited GC cell autophagy and enhanced GC cell proliferation, migration, invasion, and glycolysis *in vitro*. Further research has shown that circUBE2Q2 regulates GC advancement via the circUBE2Q2/miR-370-3p/STAT3 axis and encourages GC metastasis via exosomal communication, ultimately resulting in the malignant development of GC (72). Song et al. revealed that the exosomal hsa_circ_0017252 secreted by GC cells interfered with GC EMT by increasing E-cadherin expression and inhibiting N-calmodulin and vimentin protein production. Hsa_circ_0017252 also reduced the expression levels of IL-10 and IL-1β in macrophages and inhibited DUSP2 by upregulating inhibitor p-STAT3 expression, thereby reducing macrophage M2-like polarization to inhibit GC cell invasion and migration. Exosomal hsa_circ_0017252 inhibited GC proliferation by reducing macrophage M2-like polarization to suppress the growth of gastric tumor mass and volume *in vivo*. Mechanistically, hsa_circ_0017252 acts as a miR-17-5p sponge to inhibit GC proliferation and migration (73). Zhang et al. found that circDIDO1 overexpression prevented GC EMT, upregulating E-cadherin and significantly downregulating N-calmodulin. CircDIDO1 overexpression prevented GC growth and metastasis *in vivo*, while circDIDO1 knockdown increased GC cell migration and invasion *in vitro*. CircDIDO1 also functioned as a PARP1 inhibitor by encoding the DIDO1-529aa protein, which also promoted RBX1-mediated ubiquitination and PRDX2 degradation (74). This team’s subsequent research discovered that circDIDO1 was concentrated in RGD-modified exosomes and that it prevented the development of GC by regulating the miR1307-3p/SOSC2 axis (75). Similarly, Liang et al. found that GC stem cell (GCSC)-derived exosomal circ670 significantly promoted GCSC stemness and EMT, thereby triggering GC development. Interestingly, this study also discovered that circ670 was highly expressed in GC tissues, especially in GC patients with a history of smoking, whose circ670 expression levels were significantly increased (76), suggesting that tobacco smoke may promote the expression of circ670 in GCSC and their exosomes, which provides new insights into the molecular mechanisms by which tobacco smoke promotes GC development. In conclusion, the above studies demonstrate that circRNAs can regulate GC cell EMT and other molecular signaling pathways via exosomal secreted by GC cells, thereby promoting GC invasion and migration.

### 4.3 Exosomal circRNAs mediate GC angiogenesis

Tumor angiogenesis is a critical cause of rapid tumor proliferation, early metastasis, and poor prognosis (66). Through angiogenesis, tumor cells not only receive sufficient oxygen and nutrients but also remove carbon dioxide and metabolic waste, thus facilitating tumor cell growth and metabolism (95, 96). Tumor angiogenesis requires the synergistic cooperation of tumor cells and tumor stromal cells and their secretagogues, such as cytokines and extracellular vesicles (97). Several protein tyrosine kinase receptors are involved in tumor angiogenesis, among which vascular endothelial growth factor (VEGF) and vascular endothelial growth factor receptor (VEGFR) can promote vascular endothelial formation and have an important role in regulating tumorigenesis, development (98, 99). Bevacizumab, a clinically used tumor-targeting inhibitor, inhibits tumor angiogenesis by significantly reducing the expression of VEGF (100).

Numerous studies have reported that exosomal circRNAs may be involved in tumor angiogenesis by regulating secreted factors such as VEGF and signaling pathways, thereby influencing the development of GC. For example, Xie et al. found that circSHKBP1 expression was increased in serum exosomes and tumor tissues of GC patients. Microtubule formation assays showed that exosomal circSHKBP1 promoted VEGF secretion and induced angiogenesis, and that bevacizumab inhibited this process. In addition, serum exosomal circSHKBP1 overexpression can promote GC cell proliferation, migration, and invasion. Mechanistically, circSHKBP1 increases HUR expression and enhances VEGF mRNA stability through the adsorption of miR-582-3p. Furthermore, circSHKBP1 directly binds to HSP90 and blocks the interaction of STUB1 with HSP90, inhibiting the ubiquitination of HSP90 and thus accelerating GC development (77). Similarly, another study demonstrated that exosomal circ29 (circ_0044366) was highly expressed in GC cells and plasma of GC patients. Overexpression of exosomal circ29 significantly increased angiogenesis and mechanistically, exosomal circ29 as a miR-29a sponge to regulate VEGF expression levels in human umbilical vein endothelial cells (HUVEC), thereby promoting an aggressive phenotype of HUVEC. Moreover, Edu and Transwell assays showed that overexpressed exosomal circ29 promoted proliferation, invasion, and malignant phenotype of HUVEC (78). You et al. found by microtubule formation assay that GC cell exosomal circ_0001789 could increase the expression of VEGF-A and promote angiogenesis in endothelial cells, thus favoring the malignant progression of GC. In addition, GC cell exosomal circ_0001789 can mediate intercellular signaling exchange and induce changes in EMT markers, resulting in upregulation of N-calmodulin and wave protein expression and downregulation of E-cadherin expression. Mechanistically, circ_0001789 promotes GC genesis and metastasis by regulating the miR-140-3p/PAK2 axis (79). In summary, the above studies in the literature reveal that exosomal circRNAs regulate tumor angiogenesis and intervene in GC development as well as the malignant progression through various functional experiments and *in vivo*.

### 4.4 Exosomal circRNAs modulate GC metastasis

GC metastasis is the migration of GC cells from their primary site to other sites, is associated with multiple oncogenes, and involves multiple signaling pathways. GC metastasis is also a major cause of rapid GC progression and poor prognosis (101).

Exosomal circRNAs play a crucial role in the interaction between GC cells and other cells, contributing to GC invasion and metastasis. For example, Shen et al. found that hsa_circ_0000437 was enriched in exosomes secreted by GC cells and transported to lymphatic endothelial cells via exosomes, and gain-of-function and loss-of-function experiments showed that highly expressed exosomal hsa_circ_0000437 promoted invasion and migration of human lymphatic endothelial cells (HLECs); in a popliteal LNM model, exosomal hsa_circ_0000437 promoted lymphangiogenesis and LNM. Mechanistically, exosomal hsa_circ_0000437 induced GC LNM through a non-VEGF-C-dependent HSPA2-ERK signaling pathway. Hsa_circ_0000437 was also found to regulate GC cell apoptosis by targeting SRSF3 and inhibiting programmed cell death 4 (PDCD4), and thus by regulating SRSF3/PDCD4 axis to promote GC progression (80). Similarly, Zhang et al. showed by FISH analysis that circSTAU2 was mainly located in the cytoplasm and significantly down-regulated in GC. It was confirmed that exosomal circSTAU2 overexpression inhibited GC cell proliferation, migration, and invasion *in vivo*, and mechanistically, exosomal circSTAU2 inhibited GC progression by regulating the miR-589/CAPZA1 axis. Interestingly, a study found that CAPZA1 was negatively correlated with markers of EMT (81) and that CAPZA1 overexpression inhibited the GC EMT process, suggesting that exosomal circSTAU2 is closely associated with the development of EMT, but further confirmation of the specificity is needed. MBNL1, an RBP upstream of circSTAU2, was also found to significantly promote circSTAU2 expression (82). Another study showed that circ-RanGAP1 expression was significantly upregulated in tumor tissues and plasma exosomes of GC patients. Inhibition of exosomal circ-RanGAP1 promotes invasion of GC cells *in vitro* and vivo. Mechanistically, circ-RanGAP1 acts as a ceRNA to inhibit miR-877-3p and increase the expression of the target gene VEGFA, thereby promoting GC invasion and metastasis (83). Similarly, Sang et al. demonstrated through *in vivo* and *in vitro* functional experimental studies that the exosomal circRELL1 significantly inhibited the proliferation, migration, invasion, and anti-apoptotic capacity of GC cells and attenuated GC lung metastasis. Mechanistically, circRELL1 functions as a ceRNA at the post-transcriptional level by sponging miR-637, down-regulating EPHB3, and regulating GC autophagy activation, thereby inhibiting GC progression. Furthermore, this study confirmed that plasma exosomes maintain some stability when stored for different periods in a room-temperature environment and during repeated freeze-thaw cycles, suggesting the potential of plasma exosomal circRELL1 as a diagnostic biomarker for GC (84). Taken together, the above studies confirmed *in vivo* and *in vitro* that exosomal circRNAs act on GC cells through multiple pathways to promote or inhibit GC invasion and metastasis.

### 4.5 Exosomal circRNAs regulate GC chemotherapy resistance and radiosensitivity

Chemotherapy resistance is a key impediment to GC treatment, not only affecting GC outcomes but also leading to poor GC prognosis. Exosomes can transport a variety of drug-resistant biomolecules, including circRNAs, which regulate GC chemoresistance. For example, circ_0063526 expression is increased in GC tissues, cells, and CDDP-resistant cells. circ_0063526 promotes CDDP resistance in CDDP-sensitive GC cells via exosomal packaging. Knockdown of exosomal circ_0063526 attenuates CDDP resistance by inhibiting GC cell migration, invasion, and autophagy. Mechanistically, exosomal circ_0063526 acts as a miR-449a sponge to upregulate SHMT2 expression to enhance CDDP resistance in GC cells. Furthermore, high expression of serum exosomal circ_0063526 in GC patients correlates with CDDP resistance and adverse effects of CDDP therapy in GC patients, and serum exosomal circ_0063526 may serve as a promising prognostic biomarker for GC patients (85). Yao et al. revealed that exosomal circ-PVT1 was expressed at elevated levels in CDDP-resistant GC cells and serum, and knockdown of circ-PVT1 inhibited CDDP resistance by inducing apoptosis and inhibiting invasion or autophagy in CDDP-resistant GC cells. Mechanistically, exosomal circ-PVT1 regulates GC cell apoptosis, invasion, and autophagy through the miR-30a-5p/YAP1 axis and promotes CDDP resistance. In addition, exosomal circ-PVT1 expression levels were elevated in GC tissues, and high expression of circ-PVT1 was positively correlated with tumor LNM grade, LNM, and tumor size (86). Similarly, another study found that circ-LDLRAD3 was highly expressed in CDDP-resistant GC tissues and cells. circ-LDLRAD3 was transported by exosomes and overexpressed in CDDP-resistant GC cell-derived exosomes. circ-LDLRAD3 knockdown reduced drug resistance and inhibited the growth and invasion of CDDP-resistant GC cells, and mechanistically, knockdown of circ-LDLRAD3 inhibits CDDP chemoresistance and suppresses CDDP-resistant GC via miR-588 enrichment-mediated SOX5 (87). Liu et al. demonstrated that circ_0000260 expression was increased in tissues and serum-derived exosomes from gastric adenocarcinoma patients. Circ_0000260 was more highly expressed in CDDP-resistant gastric adenocarcinoma cells compared to CDDP-sensitive gastric adenocarcinoma cells *in vitro*. Circ_0000260 knockdown increased CDDP chemotherapy sensitivity, inhibited CDDP-resistant gastric adenocarcinoma cells proliferation, migration, invasion, and adhesion, and induced apoptosis. Circ_0000260 knockdown reduced CDDP chemoresistance and inhibited tumor growth *in vivo*, and mechanistically, circ_0000260 acted as a ceRNA to regulate gastric adenocarcinoma development and CDDP resistance by targeting mir-129-5p, which upregulates MMP11 progression (88). Chen et al. revealed that circ_0091741 is highly expressed in GC cells and their secreted exosomes, and mechanistically, GC cell-derived exosomal circ_0091741 competitively binds miR-330-3p, decreasing its binding to the target gene TRIM14, thereby increasing TRIM14 expression, and TRIM14 overexpression promotes GC cell autophagy and oxaliplatin (OXA) resistance, Furthermore, TRIM14 can activate the Wnt/β-catenin signaling pathway by stabilizing Dvl2, thus exosomal circ_0091741 promotes GC cell autophagy and chemoresistance through the miR-330-3p/TRIM14/Dvl2/Wnt/β-catenin axis (89). He et al. found that exosomal circPRRX1 interfered with GC cell proliferation, migration, invasion, and radiosensitivity by MTT, cell colony formation, and transwell assays, and that mechanistically, exosomal circPRRX1 acted as a ceRNA for miR-596 to upregulate NF-κB activating protein (NKAP) thereby regulating GC development and radiosensitivity (90). Taken together, the above findings suggest that GC cells remodel the GC TME via exosomal circRNAs to regulate resistance to chemotherapeutic agents as well as radiosensitivity.

## 5 Potential of exosomal circRNAs in the diagnosis and prognosis of GC

Currently, the biomarkers used to screen for GC such as glycoprotein chain antigen 125 (CA125), CA19-9, CA72-4, pepsinogen I/II ratio (PG I/PG II), gastrin-17 (G-17), anti-gastric parietal cell antibodies (APCA), squamous cell carcinoma antigen (SCCA), cytokeratin 19 fragment (CYFRA 21-1), and carcinoembryonic antigen (CEA) are found to be not sensitive and specific enough during the time of diagnosis, as reported by studies (3, 102, 103). Therefore, it is imperative to continue searching for novel biomarkers that can aid in the early detection of GC.

Several studies have shown that GC-derived or GC-associated exosomal circRNAs are closely associated with the TNM stage, tumor volume, survival, and poor prognosis of GC and that the expression of exosomal circRNAs at different stages of GC has high sensitivity and specificity. As a non-invasive marker, exosomal circRNAs may become a reliable indicator for GC diagnosis and prognosis **Table 2**. For example, circFCHO2 is upregulated in serum exosomes of GC patients. The receiver operating characteristic (ROC) curve suggests that serum exosomal circFCHO2 is a sensitive and effective diagnostic indicator of GC, with the area under the ROC curve (AUC) of 0.8449. CircFCHO2 was more highly expressed in GC patients with LNM and pulmonary metastases compared to patients without LNM. Moreover, circFCHO2 expression correlated with poor survival in GC patients. GC patients with high circFCHO2 expression had shorter metastasis-free survival, suggesting that high circFCHO2 expression may be a marker of poor prognosis in GC (69). Similarly, another study found that the exosomal hsa_circ_0000437 was enriched in the serum of GC patients and significantly correlated with the TNM stage of GC and with LNM. The ROC curve analysis showed that serum exosomal hsa_circ_0000437 could discriminate between GC patients and healthy volunteers with the AUC of 0.808, indicating that serum exosomal hsa_circ_0000437 has high diagnostic accuracy. The Kaplan-Meier survival curve (KM) showed that high expression of serum exosomal hsa_circ_0000437 was associated with poor survival outcomes in GC patients, suggesting that serum exosomal hsa_circ_0000437 may be a diagnostic and prognostic biomarker for GC (80). Shi et al. found that circ_0088300 expression was significantly higher in plasma exosomes of GC patients and was consistent with levels in GC tissues. The ROC curve showed that plasma exosomal circ_0088300 has a high reference value in the diagnosis of GC with the AUC of 0.7961; the survival curve suggested that plasma exosomal circ_008830 survival was shorter in GC patients with high expression than in GC patients with low expression, suggesting that plasma exosomal circ_0088300 may serve as a diagnostic and prognostic biomarker for GC (67). Another study showed that circ-RanGAP1 expression was elevated in plasma exosomes of preoperative GC patients. High expression of circ-RanGAP1 was associated with tumor volume, TNM stage, LNM, and poor prognosis in GC patients. Prognostic models showed that plasma exosomal circ-RanGAP1 combined with TNM staging was more effective in assessing GC prognosis, with the AUC of 0.830 compared to 0.646 for plasma exosomal circ-RanGAP1 and 0.779 for TNM staging. In addition, plasma exosomal circ-RanGAP1 can be used to differentiate GC patients from healthy individuals and may be used as a diagnostic marker for GC (83). The expression of circ-KIAA1244 was reduced in GC tissues, cells, plasma, and plasma exosomes. Low expression of plasma exosome circ-KIAA1244 was negatively correlated with GC TNM stage and LNM. The ROC curve showed that circ-KIAA1244 differentiated GC patients from normal healthy individuals, with the AUC of 0.7481, a sensitivity of 77.42%, and a specificity of 68.00%. The KM showed that patients with low expression of circ-KIAA1244 had shorter survival times. These results suggest that plasma exosomes circ-KIAA1244 may be an independent prognostic indicator for GC patients (104). Similarly, another study found that hsa_circ_0015286 was highly expressed not only in GC cell-derived exosomes but also in GC patients’ tissues and plasma exosomes. Compared to chronic gastritis and normal healthy volunteers, plasma exosomal hsa_circ_0015286 was expressed at significantly increased levels in GC patients and was strongly correlated with tumor size, TNM stage, and LNM in GC patients. The combination of exosomal hsa_circ_0015286 with CEA and CA19-9 has a higher value for the diagnosis of GC, with the AUC of 0.843, compared to 0.778, 0.673, and 0.665 respectively when tested alone. The expression level of exosomal hsa_circ_0015286 decreased significantly in GC patients after surgical excision of the lesion, and GC patients with low expression of exosomal hsa_circ_0015286 had a significantly longer overall survival than those with high expression of exosomal hsa_circ_0015286. Exosomal hsa_circ_0015286 may be a potential biomarker for monitoring dynamic changes in GC and prognosis (105). In short, the above studies in the literature show that exosomal circRNAs have higher sensitivity and specificity in the diagnosis of GC and may become a specific biomarker for GC diagnosis and prognosis in the clinic in the future.

**Table 2:** The potential value of exosomal circRNAs in the diagnosis and prognosis of GC.

circRNAs	Source	Expression	isolation methods	Biomarker	AUC	Sensitivity	Specificity	Ref
circ_0088300	Plasma	Up	Ultracentrifugation	Diagnosis and prognosis	0.7961	–	–	(67)
circFCHO2	Serum	Up	Isolation kit	Diagnosis and prognosis	0.8449	–	–	(69)
hsa_circ_0000437	Serum	Up	Differential centrifugation	Diagnosis and prognosis	0.808	–	–	(80)
circ-RanGAP1	Plasma	Up	Ultracentrifugation	Diagnosis and prognosis	0.646	–	–	(83)
circRELL1	Plasma	Down	Ultracentrifugation	Diagnosis	0.731	–	–	(84)
circ_0063526	Serum	Up	ExoQuick precipitation kit	prognosis	–	–	–	(85)
circ-KIAA1244	Plasma	Down	Hieff™ Quick Isolation kit	Diagnosis and prognosis	0.7481	77.42%	68.00%	(104)
hsa_circ_0015286	Plasma	Up	ExoQuick solution	Diagnosis and prognosis	0.778	0.821	0.657	(105)

## 6 Potential of exosomal circRNAs in targeted therapy of GC

As chemotherapy resistance and its associated side-effects continue to increase in cancer treatment, targeted therapies have become an effective complementary treatment (106). Considering the biological functions of exosomal circRNAs and their role in GC proliferation, invasion, and metastasis, exosomal circRNAs could be a potential target for GC therapy in addition to being molecular markers for GC diagnosis and prognosis.

**Figure 3:**
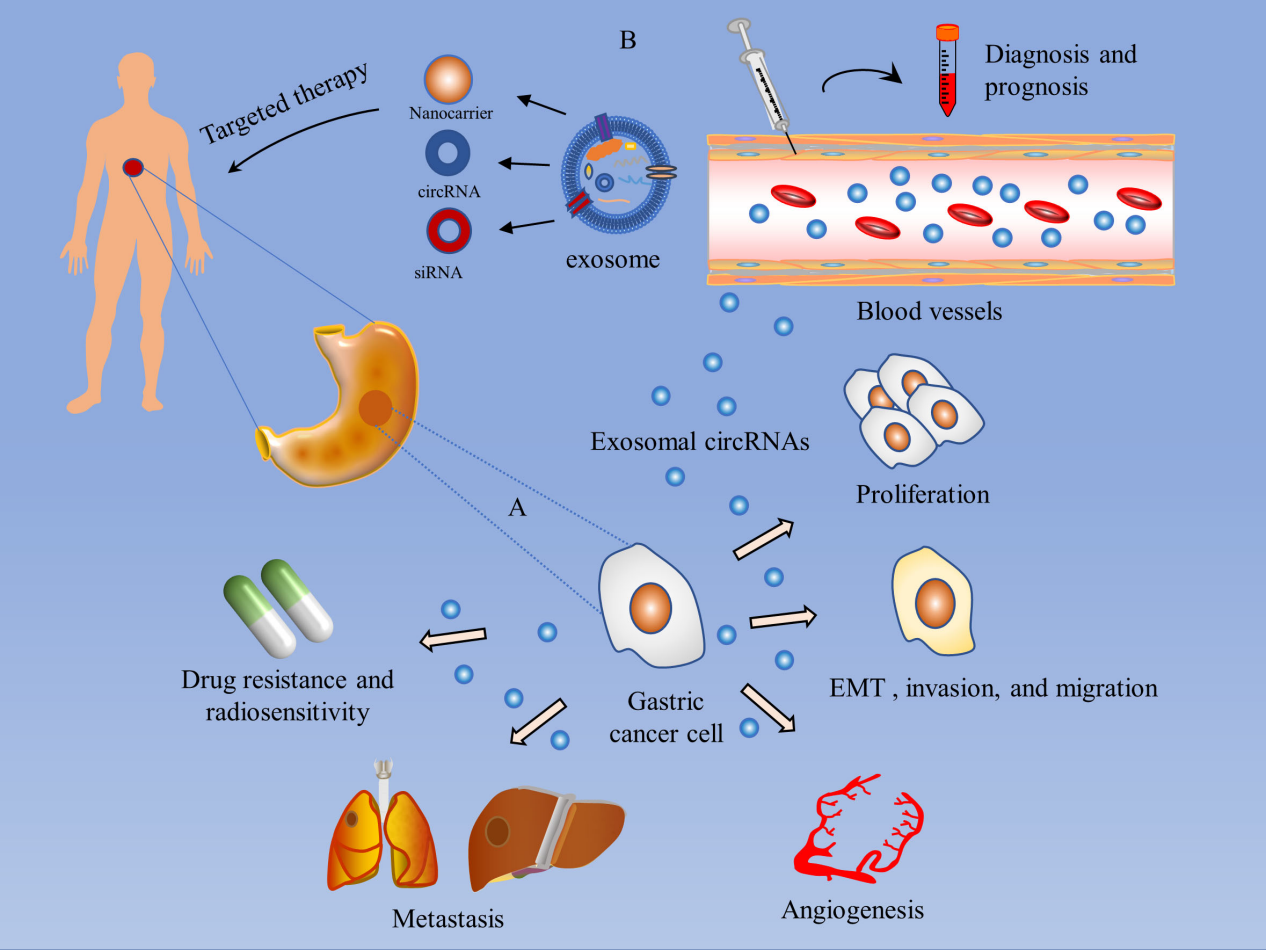
Mechanism of action of exosomal circRNAs in GC and their value in GC diagnosis and treatment. **(A)** Mechanisms of action of exosomal circRNAs in GC: 1) Promotion of GC proliferation; 2) Induction of EMT and invasion and migration of GC cells; 3) Mediation of GC angiogenesis; 4) Regulation of GC metastasis; 5) Modulation of chemotherapy resistance and radiosensitivity in GC. **(B)** The value of exosomal circRNAs in the management of GC: 1) Diagnostic and prognostic assessment of gastric cancer patients by detection of serum or plasma exosomal circRNAs. 2) Targeted drugs to treat gastric cancer through exosomal delivery of small interfering RNAs (siRNAs), or to intervene in GC progression by modifying exosomes to load anti-cancer circRNAs.

As mentioned above, circRNAs can act as drivers or inhibitors of GC. Intervening with circRNAs through effective technical means of gene overexpression or knockdown may provide clues to targeted therapies for GC. Exosomes naturally transport a variety of substances and are used to deliver circRNAs with specific therapeutic effects to the corresponding GC cells, thereby producing therapeutic effects. For example, small interfering RNAs (siRNAs) are designed to target specific circRNAs and delivered to GC cells via exosomes to help reduce the expression of oncogenic circRNAs in cancer cells (107), or to intervene in GC progression by modifying exosomes to load anti-cancer circRNAs into target cells. Guo et al. found that delivery of circDIDO1 via RGD-modified exosomes (RGD-Exo-circDIDO1) to GC cells inhibited their proliferation, migration, and invasion, and promoted apoptosis. GC mice model treated with RGD-Exo-circDIDO1 showed no significant pathological lesions in the kidney, spleen, heart, liver, and lung tissues, and no significant abnormalities in liver and kidney function, indicating that RGD-Exo-circDIDO1 is safe and effective in the treatment of GC and may be a practical treatment for GC (75). Therefore, exosome engineering based on circRNAs may be an effective strategy for GC treatment in the clinic. In addition, given that exosomes are biocompatible and well-targeted and do not interfere with the pharmacological effects of drugs, exosomes hold great promise as drug delivery vehicles in the treatment of cancer (17, 108) (**Figure 3**).

## 7 Discussion and perspectives

Gastric cancer is a common malignancy of the digestive system and a leading cause of cancer-related deaths (7). Although surgical treatment, radiotherapy, targeted therapy and immunotherapy provide additional treatment options for gastric cancer patients, the therapeutic effects for patients with advanced gastric cancer are still restricted (3). Molecular typing of gastric cancer provides an opportunity for individualized treatment of gastric cancer patients, especially markers such as microsatellite instability (MSI), programmed cell death ligand (PD-L1), human epidermal growth factor receptor 2 (HER2), tumor mutation burden (TMB) and mismatch repair deficiency (dMMR) can select gastric cancer patients who would benefit from immunotherapy and targeted therapy. However, the existing markers have low positivity rates in gastric cancer patients, which to some extent restricts the personalized diagnosis and treatment of gastric cancer. Therefore, the continued search for effective biomarkers and potential therapeutic targets is of great importance for the clinical diagnosis and treatment of gastric cancer (6). Currently, tumor treatment has shifted from targeting tumor cells to regulating TME, making TME a potential target for gastric cancer treatment. TME is an essential prerequisite for tumor development, and among the many components of TME, exosomal circRNAs are an indispensable part. and exosomal circRNAs are a hotspot in the field of oncology research at present. Exosomal circRNAs can regulate EMT, angiogenesis, tumor metabolism, drug resistance, tumor proliferation, invasion, and metastasis. This review summarizes the literature on the mechanisms of exosomal circRNAs in GC and finds that the mechanisms of exosomal circRNAs in GC are not fully elucidated, and most of the studies focus on exosomal circRNAs as a ceRNA network that sponge miRNAs to regulate the expression of target proteins and thus influence the malignant progression of GC. Furthermore, the regulation of the immune microenvironment in GC has been relatively understudied, and the majority of existing studies have focused on tumor proliferation, invasion and metastasis, and drug resistance in GC. Therefore, the role of exosomal circRNAs in the immune microenvironment of GC and their mechanisms can be further explored in the future. With the development and advancement of exosome engineering technology, the identification of circRNAs that can affect PD-1/PD-L1 expression in GC cells and their transfer to target cells through engineering-engineered exosomes, thereby increasing the sensitivity of targeted drug therapy, holds great potential for targeted therapy in GC.

CircRNAs are enriched in exosomes and have good stability, which would facilitate the storage, isolation, and detection of circRNAs. Exosomes are widely present in the body fluids of GC patients, especially in the patients’s blood (109). Therefore, the detection of exosomal circRNAs by non-invasive methods to screen early GC patients and develop personalized treatment plans will enable GC patients to receive timely and effective treatment and achieve better prognostic outcomes. Liquid biopsy is of great value for screening exosomal circRNAs due to its non-invasive, low-cost, high-efficiency, and high-precision features. Previous studies have shown that upper gastrointestinal endoscopic biopsy is the gold standard for GC diagnosis (103). However, it is an invasive operation and can lead to undesirable complications. Tissue biopsy is affected by tumor heterogeneity, which can easily lead to unsuccessful biopsies. As tumor research has become more refined, the move towards a molecular analysis model of biological fluids has made liquid biopsies more advantageous in clinical practice. Compared to tissue biopsy, liquid biopsy is less invasive, less expensive, and relatively less affected by tumor heterogeneity, while liquid biopsy allows access to the spectrum of variability present in heterogeneous tumors and real-time monitoring of changes in tumor status (41). For example, by detecting circulating tumor DNA (ctDNA), and circulating tumor cells (CTCs) in the blood, it is possible to assess microscopic tumor foci that remain after tumor resection (6). Although ctDNA and CTCs have made great progress in the application of liquid biopsy for monitoring tumor recurrence and metastasis, exosomal circRNAs may be a useful complement and have a broader potential for application. The application of liquid biopsy techniques to detect exosomal circRNAs is of great importance for early diagnosis and therapeutic stratification in GC. However, there is currently no method that can guarantee the purity, content, and biological activity of isolated and extracted exosomes. Ultracentrifugation is a common method for the isolation and extraction of exosomes (110). However, it does not exclude other components of similar volume and density in the sample and is complex and time-consuming; immunoaffinity capture has limited purity due to the absence of specific markers on the exosome surface, and other methods including exosome extraction kits have corresponding drawbacks and cannot guarantee the purity of exosomes (111). The limitations of exosome isolation and purification methods are also a major factor limiting exosome-related research, so further improvement of exosome isolation and purification methods and the establishment of a uniform and standardized technique will enable more effective application of exosomal circRNAs in clinical practice.

Exosomal circRNAs have the potential to become GC-targeted therapeutics, but many issues remain to be addressed, including the route of exosome delivery when using exosomes as a vehicle for therapy, the site of delivery, the specific quantitative-effect relationship when exosomes are loaded with circRNAs, the timing of intervention in GC therapy, and the stability of exosomal circRNAs under conditions of light and different temperature gradients still need to be further addressed (112, 113). Preclinical models are also needed to assess the safety of exosomal circRNAs treatment and to test their pharmacological toxicity *in vivo* and *in vitro*. Furthermore, the actual translation of exosomal circRNAs into clinical applications needs to be validated by large, multicenter prospective cohort studies. Therefore, although exosomal circRNAs have a promising future in the clinical management of GC, there is still a long way to go before they can become diagnostic and prognostic biomarkers and therapeutic targets for GC and other tumors.

## 8 Conclusion

In conclusion, exosomal circRNAs play a significant role in the incidence and progression of GC, and they may also serve as key diagnostic and prognostic biomarkers as well as therapeutic targets for GC. It will be more beneficial for the clinical translation application of exosomal circRNAs as a result of the ongoing development and enhancement of the technology of exosomal circRNAs isolation and extraction. The range of applications for exosomal circRNAs in GC will be further broadened by continuing in-depth research on the molecular mechanisms of exosomal circRNAs in the TME. It is believed that with the continuous progress of exosomal circRNAs research, exosomal circRNAs will be applied in clinical practice in the future.

## Author contributions

SF guided the direction of this manuscript. FL designed the review and provided supervision. MZ, MQ, and YZ collected relevant literature. HM drafted and corrected the manuscript. All of the authors have read and approved the final manuscript.

## Glossary

APCA: anti-gastric parietal cell antibodies
AUC: an area under the ROC curve
CAF: cancer-associated fibroblasts
CA125: carbohydrate antigen 125
CDDP: cisplatin
CEA: carcinoembryonic antigen
CeRNA: competitive endogenous RNA
CircRNAs: Circular RNAs
CiRNAs: intron circRNAs
CTCs: circulating tumor cells
CtDNA: circulating tumor DNA
CYFRA 21 -1: cytokeratin 19 fragment
EcircRNAs: exon circRNAs
EIciRNAs: exon-intron circRNAs
EMT: epithelial mesenchymal transition
ER: endoplasmic reticulum
ESCRT: endosomal sorting complex required for transport
G-17: gastrin-17
GC: Gastric cancer
GCSC: Gastric cancer stem cells
Golgi: Golgi apparatus
HnRNPs: heterogeneous nuclear ribonucleoproteins
HUVEC: human umbilical vein endothelial cells
HLECs: human lymphatic endothelial cells
LncRNAs: long non-coding RNAs
LNM: lymph node metastasis
MVBs: multivesicular vesicles
ILVs: intraluminal vesicles
MiRNAs: microRNAs
MRNAs: messenger RNAs
NcRNAs: non-coding RNAs
NKAP: NF-KB activating protein
PDCD4: programmed cell death 4
PG I/PG II: pepsinogen I/pepsinogen II ratio
PM: plasma membrane
ROC: Receiver operating characteristic
SCCA: squamous cell carcinoma antigen
SiRNAs: small interfering RNAs
TEX: Texosome
RBPs: RNA-binding proteins
TME: tumor microenvironment
VEGF: vascular endothelial growth factor
VEGFR: vascular endothelial growth factor receptor
